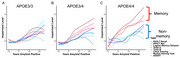# Cognitive Trajectories in Alzheimer's Disease Differ in APOE4 Carriers

**DOI:** 10.1002/alz70857_098712

**Published:** 2025-12-24

**Authors:** Casey R Vanderlip

**Affiliations:** ^1^ University of California, Irvine, Irvine, CA, USA

## Abstract

**Background:**

Apolipoprotein E4 (APOE4) is the strongest genetic risk factor for sporadic Alzheimer's disease (AD) and is linked to earlier and more extensive amyloid‐beta (Aβ) deposition. Nearly all individuals homozygous for APOE4 show elevated Aβ levels by age 80, and these individuals often experience more pronounced episodic memory deficits compared to non‐carriers. Here, we investigated whether cognitive trajectories differ by APOE status and explored whether the heightened cognitive decline in APOE4 carriers stems from increased Aβ deposition or greater sensitivity to Aβ‐related effects.

**Method:**

Using data from the Alzheimer's Disease Research Initiative, we modeled amyloid duration (i.e., the estimated number of years an individual has been Aβ+) and examined its impact on cognitive trajectories across a range of neuropsychological assessments. These assessments measured episodic memory, processing speed, executive function, visuospatial skills, language, and crystallized intelligence.

**Result:**

Our findings demonstrate that APOE4 is associated with a more rapid decline in episodic memory as a function of amyloid duration, with APOE4 homozygotes showing faster declines than heterozygotes. This decline began almost immediately after Aβ positivity was reached and was more pronounced compared to non‐carriers. Notably, this pattern was specific to episodic memory and was not observed in the other cognitive domains assessed.

**Conclusion:**

These findings indicate that cognitive trajectories in AD vary by APOE genotype. Future studies should explore whether these differences reflect distinct pathological mechanisms in APOE4 carriers.